# Pennsylvania

**Published:** 1897-04

**Authors:** 


*Pennsylvania.* One herd in the northwestern part of the State, numbering 171, proved to have 157 tuberculous. This herd had no known cases seven and a half years ago, when seven head of short-horn cattle were purchased from a New York herd, most of which were in a bad condition at the time, as they were coughing, and many soon died. Since then many deaths have occurred. The average age of the herd was a little over four and a half years. Surely no one will dispute the need of laws to protect our dairy interests, the only profitable business to the farmer in Pennsylvania, from such disastrous results.

---

Dr. Emil Knight reports a two-year old cat presented for castration, with but one descended testicle. This was removed, and though twelve months have elapsed the second one has not made its appearance in the scrotum; the feline still frequents his old haunts on stated occasions.

